# Pharmacokinetics and Drug-Drug Interactions of Abacavir and Lamuvudine Co-administered With Antituberculosis Drugs in HIV-Positive Children Treated for Multidrug-Resistant Tuberculosis

**DOI:** 10.3389/fphar.2021.722204

**Published:** 2021-10-08

**Authors:** Louvina E. van der Laan, Anthony J. Garcia-Prats, H. Simon Schaaf, Jana L. Winckler, Heather Draper, Jennifer Norman, Lubbe Wiesner, Helen McIlleron, Paolo Denti, Anneke C. Hesseling

**Affiliations:** ^1^ Department of Paediatrics and Child Health, Desmond Tutu TB Centre, Faculty of Medicine and Health Sciences, Stellenbosch University, Cape Town, South Africa; ^2^ Department of Medicine, Division of Clinical Pharmacology, University of Cape Town, Cape Town, South Africa; ^3^ Department of Pediatrics, Divisions of General Pediatrics and Adolescent Medicine and Global Pediatrics, University of Wisconsin School of Medicine and Public Health, Madison, WI, United States

**Keywords:** tuberculosis, paediatrics (drugs and medicines), HIV, drug drug interactions, pharmacometric modeling

## Abstract

Given the high prevalence of multidrug-resistant (MDR)-TB in high HIV burden settings, it is important to identify potential drug-drug interactions between MDR-TB treatment and widely used nucleoside reverse transcriptase inhibitors (NRTIs) in HIV-positive children. Population pharmacokinetic models were developed for lamivudine (n = 54) and abacavir (n = 50) in 54 HIV-positive children established on NRTIs; 27 with MDR-TB (combinations of high-dose isoniazid, pyrazinamide, ethambutol, ethionamide, terizidone, fluoroquinolones, and amikacin), and 27 controls without TB. Two-compartment models with first-order elimination and transit compartment absorption described both lamivudine and abacavir pharmacokinetics, respectively. Allometric scaling with body weight adjusted for the effect of body size. Clearance was predicted to reach half its mature value ∼ 2 (lamivudine) and ∼ 3 (abacavir) months after birth, with completion of maturation for both drugs at ∼ 2 years. No significant difference was found in key pharmacokinetic parameters of lamivudine and abacavir when co-administered with routine drugs used for MDR-TB in HIV-positive children.

## Principal Investigator Statement

The authors confirm that the PI’s for this paper are Prof A C Hesseling and Prof H S Schaaf. They had direct clinical responsibility for the patients.

### What is Already Known About the Study?

• The burden of multidrug-resistant (MDR)-TB (*Mycobacterium tuberculosis* resistant to rifampicin and isoniazid) in children is increasing. In high HIV-burden settings, a substantial proportion (20–53.9%) of these children are HIV co-infected.
• The World Health Organization (WHO) recommends lamivudine and abacavir as preferred dual nucleoside reverse transcriptase inhibitors for initial antiretroviral treatment in HIV-positive children older than 3 months of age.
• There is no data on the potential effect of MDR-TB drugs on lamivudine and abacavir in children.

### What this Study Adds:

• The study describes the pharmacokinetics of lamivudine and abacavir in HIV-positive children with and without MDR-TB treatment and could detect no significant drug-drug interactions of MDR-TB treatment on lamivudine and abacavir.
• Despite modest numbers, our results are reassuring for the antituberculosis drugs used commonly in children including terizidone, ethambutol, ethionamide, high-dose isoniazid, pyrazinamide, amikacin and fluoroquinolones as a group. For individual drugs, such as moxifloxacin, levofloxacin, linezolid and *para*-aminosalicylic acid (PAS), definitive conclusions were not possible given our small numbers and no patients were on bedaquiline and delamanid.

## Introduction

The burden of multidrug-resistant (MDR)-TB (*Mycobacterium tuberculosis* resistant to rifampicin and isoniazid) is increasing, with modelled estimates of 25,000−32,000 incident cases annually in children (Jenkins and Yuen, 2018). In high HIV-burden settings, a substantial proportion (20–53.9%) of these children are HIV co-infected (Fairlie et al., 2011; Seddon et al., 2011; Hesseling et al., 2012). HIV co-infection is associated with higher morbidity and mortality in children with MDR-TB (Seddon et al., 2011; Seddon et al., 2014). Optimizing both HIV and TB treatment in children, is critically important.

Data regarding the impact of MDR-TB drugs on the pharmacokinetics of antiretroviral drugs (ARVs) are limited, especially in children. A recent report found no significant effect of older MDR-TB drugs on lopinavir-ritonavir exposures in HIV-positive children (Van der Laan et al., 2018). The effect of MDR-TB drugs on nucleoside reverse transcriptase inhibitors (NRTIs) in children has not yet been studied.

Data on the potential effect of MDR-TB drugs on lamivudine and abacavir, the World Health Organization (WHO)-recommended preferred dual NRTI backbone for initial antiretroviral treatment (ART) in HIV-positive children older than 3 months of age, is particularly relevant (World Health Organization, 2018).

Lamivudine is widely distributed into total body fluid which may be partly related to its low protein binding (generally <36%) (Perry and Faulds, 1997; Johnson et al., 1999). Protein binding for abacavir is around 50% and it is suggested that the drug is distributed to extravascular spaces (Yuen et al., 2008). Lamivudine undergoes minimal metabolism and is primarily renally eliminated (approximately 70%) (Heald et al., 1996). Abacavir is extensively metabolised by the liver with less than 2% excreted unchanged in urine (Chittick et al., 1999; McDowell et al., 1999); it is primarily metabolised via two pathways, urine diphosphate glucuronyltransferase and alcohol dehydrogenase (Yuen et al., 2008). Lamivudine induces p-glycoprotein (Weiss et al., 2008), whereas abacavir is a substrate and possible inhibitor (Shaik et al., 2007; Storch et al., 2007; Namanja et al., 2012). These are all sites for possible DDIs with drugs used to treat MDR-TB.

There are a number of potential interactions with MDR-TB drugs included in this study. Clofazimine is metabolised by, and a weak inhibitor of the CYP P450 enzyme system (Cholo et al., 2012; Sangana et al., 2018). Moxifloxacin undergoes partial hepatic metabolism (Lehmann, 1969; Clofazimine, 2008; Sy et al., 2015) and primarily renal mechanisms account for the elimination of PAS, linezolid, levofloxacin, amikacin, and terizidone (Slatter et al., 2001; Garcia-Prats Donald et al., 2013). As substrates of p-glycoprotein, linezolid and moxifloxacin (Escribano et al., 2007; Brillault et al., 2009; Bolhuis et al., 2013) might interact with lamivudine and abacavir through efflux pump transport mechanisms.

Previous population pharmacokinetic models for lamivudine and abacavir in children have included one (Tremoulet et al., 2007; Piana et al., 2013) (Jullien et al., 2005) and two (Bouazza et al., 2011; Zhang et al., 2012) (Zhao et al., 2013; Rabie et al., 2020) compartment models, with first-order elimination, and either first-order absorption or transit compartments (Zhang et al., 2012; Rabie et al., 2020). These former models were used to inform our final population pharmacokinetic models with the aim to characterize the effect of routine antituberculosis drugs used for MDR-TB treatment on the pharmacokinetics of lopinavir and abacavir in HIV-positive children.

## Methods

The study was conducted in Cape Town, South Africa, as part of the MDR-PK1 study (R01 HD069169-01). The study was approved by Stellenbosch University (N11/03/059) and the University of Cape Town (397/2011) Health Research Ethics Committees. Written informed consent was obtained from parents/legal guardians and assent was obtained from participants where appropriate.

HIV-positive children (ages 0 to <15 years) routinely treated for MDR-TB were consecutively enrolled (MDR-TB group). A control group of HIV-positive children without TB was matched roughly to the MDR-TB group for age and according to use of lopinavir/ritonavir or efavirenz-based combination ART. Children were established on at least 2 weeks of lamivudine, abacavir and MDR-TB treatment prior to enrolment.

Lamivudine was given as a 4 mg/kg twice-daily or 8 mg/kg once-daily dose. Abacavir was given as an 8 mg/kg twice-daily or 16 mg/kg once-daily dose. Both drugs were given as either a suspension or tablet (whole or crushed with water). Brands included *Aspen, Aurobindo, Adcock Ingram*, *Sonke, Mylan* and *Cipla.* Some children (<2 years), that refused to swallow, were dosed using a nasogastric tube. All antituberculosis drugs were administered after a minimum of 4 h fast; ARVs including lamivudine and abacavir were dosed 1 h later and afterwards a breakfast was offered.

Since the study was nested in a larger study on MDR-TB drugs, the sampling schedule was optimised around the time of the dose of antituberculosis drugs. Blood samples were drawn at six time points: two samples before dosing ARVs [1 h (time −1 h, just before administration of the antituberculosis drugs) and immediately before (time 0) the ARV dose] and at 1, 3, 7 and either 5 or 10 h after observed dosing. For participants who remained on once-daily dosing, samples were obtained the following day, at 10, 11, 13, 15, and 17 h after the evening ARV dose.

The assays for lamivudine and abacavir were developed at the Division of Clinical Pharmacology, University of Cape Town (Archary et al., 2019). Validation was done according to the US Food and Drug Administration (Author Anonymous, 2011) and European Medicines Agency (European Medicines Agency, 2012) guidelines.

Population pharmacokinetic analyses were completed using NONMEM version 7.4.3 (Icon Development Solutions, Ellicott City, MD, United States of America). All statistical analyses, including summary statistics and visual displays, were generated in R (http://www.R-project.org/) while Perl-speaks-NONMEM (PsN), Pirana and the R package Xpose4 (http://xpose.sourceforge.net) were used in the model building process for data exploration, visualization and creation of diagnostics (Keizer et al., 2013).

Several structural models were tested for lamivudine and abacavir: one- and two-compartment disposition with first-order elimination, and first-order absorption, with and without absorption lag time or transit compartments (Savic et al., 2007).

The pharmacokinetic samples collected pre-dose were treated as a separate occasion in the model, to allow estimation of both inter-individual (IIV) and inter-occasion variability (IOV). A lognormal distribution was assumed for these random effects and correlation between them was investigated both at IIV and IOV level. The relative bioavailability was fixed to one for a typical patient to investigate the presence of IIV and/or IOV on this parameter. The residual unexplained variability (RUV) was evaluated using a combined additive and proportional model. Samples with concentrations below the limit of quantitation (BLQ) were handled by the M6 method from Beal (Beal, 2001). This means that they were replaced with half the lower limit of quantitation (LLOQ), except for consecutive values in a series, for which the trailing BLQ values were ignored for the fit but included in the diagnostic plots. The additive error was inflated by half the LLOQ value for the imputed BLQ values (i.e., by LLOQ/2) to allow for extra uncertainty due to the imputation (and proportionally to the size of the LLOQ for that specific assay) (Francis et al., 2021). Finally, the additive error for all samples obtained from a specific assay was bound to be at least 20% of the LLOQ of that assay.

Allometric scaling was applied to oral clearance, intercompartmental clearance, volume of distribution and peripheral volume of distribution to account for differences in body size (Anderson and Holford, 2008). Besides total body weight, fat-free mass (Al-Sallami et al., 2015) was also tested as alternative descriptor of body size.

After the inclusion of allometric scaling, covariate selection was performed by first narrowing down the search to factors that were either known or physiologically plausible to affect a certain pharmacokinetic parameter. Then, the plots of individual random effects (Empirical Bayes Estimates) (Lavielle and Ribba, 2016) versus covariates were used to identify possibly significant trends in the data. Finally the candidate covariate effect were tested and included in the model using a step-wise procedure with forward inclusion (*p* < 0.05 based on drop in −2 log-likelihood (-2LL)) and backward elimination (*p* < 0.01) (Wählby et al., 2015). Additionally, the improvement in goodness of fit including visual predictive checks, reduction in unexplained variability, and stability of the model parameter estimates were considered to retain the effects in the model.

Age was tested using a sigmoidal maturation model (Anderson and Holford, 2008; Holford et al., 2013), as shown in the equation below

$$MAT=\frac{PMAGE^{\gamma }}{PMAGE_{50}^{\gamma }+PMAGE^{\gamma }}$$ (1)

where MAT is the fraction of the adult value of clearance, PMAGE is postmenstrual age (post-natal plus gestational age), with $PMAGE_{50}$ being the value of PMAGE when maturation reaches 50% of the adult clearance, and  γ (also known as the Hill coefficient) is the shape factor of the curve.

MDR-TB treatment, as a single combined variable or selected single MDR-TB drugs, was used as a categorical covariate to test whether parameter estimates (oral clearance, bioavailability, and the absorption parameters including mean transit time) were different between lamivudine and abacavir in the MDR-TB group and the control group.

Other covariates tested for significance in the model were sex, method of drug administration on the sampling day (nasogastric tube vs oral, crushing tablets), drug formulation (suspension/whole tablets) and other ART treatment (lopinavir/ritonavir and efavirenz).

Model development was guided by changes in the −2LL (with drops of more than 6.64 points considered significant at *p* < 0.01 for the inclusion of one additional parameter in the model), precision in parameter estimates, graphical analysis of goodness of fit plots including visual predictive checks, and scientific plausibility (Jonsson and Karlsson, 1998; Karlsson and Holford, 2008; Kiang et al., 2012). Parameter uncertainty of the estimated from the final model was assessed using Sampling Importance Resampling (Dosne et al., 2016).

### Statistical Power Calculations

This analysis was nested in a larger study of children with MDR-TB, and with no clear expectations on the size of expected drug-drug interactions and the number of children that would have been on each specific MDR-TB drug, so no formal calculation of statistical power was prospectively performed. We however assessed *a posteriori* the statistical power of our data to detect significant differences in ART exposure between HIV-positive children with MDR-TB and controls, based on simulation and re-estimation (Lee, 2001), using the stochastic simulation and estimation (SSE) tool of Perl-speaks-NONMEM (Lindbom et al., 2004). Briefly, the final pharmacokinetic model was used to re-simulate (*n* = 200) the current trial (thus assuming the same patient covariates, doses, and sampling times), but with postulation of a known difference in clearance or bioavailability between the MDR-TB and control groups. Then, alternative models with or without this MDR-TB covariate effect were fitted to each simulated data set and compared to evaluate whether the effect was statistically significant in terms of improvement of the −2LL value. The percentage of simulated data in which the effect could be detected as significant, provided the statistical power.

## Results

A summary of participant characteristics is presented in Table 1. Fifty-four HIV-positive children (27 MDR-TB and 27 control cases) were included. All children were on both NRTIs, lamivudine and abacavir, except for two controls on zidovudine and two MDR-TB cases on stavudine as substitute for abacavir.

**TABLE 1:** Summary of characteristics of HIV-positive children with and without MDR-TB on a lamivudine and abacavir–containing antiretroviral regimen^a^.

Characteristics	Lamivudine		Abacavir	
	MDR-TB (*n* = 27)	Control (*n* = 27)	MDR-TB (*n* = 25)	Control (*n* = 25)
Median (IQR) age (yr)	4.2 (1.6–9.6)	5.7 (1.6–9.5)	2.9 (1.4–9.4)	5.8 (2.7–9.6)
No (%) of male children	12 (44)	13 (48)	11 (44)	12 (48)
Median (IQR) weight (kg)	13.4 (9.1–21.4)	15.6 (11.2–23.1)	13.2 (9.0–21.1)	16.3 (11.5–23.3)
Black ethnicity no. (%)	24 (89)	14 (52)	22 (88)	12 (48)
No (%) of children with a Z-score < -2^b^				
Weight-for-age	16 (59)	9 (33)	16 (64)	8 (32)
Length-for-age	16 (59)	10 (37)	16 (64)	9 (36)
Median (IQR) CD4^+^ T-cell count (cells/ μl )^c^	549.5 (337.8–1,210)	1,026 (637–1,535)	549.5 (326.8–1,184)	1,026 (710–1,512.5)
Median (IQR) viral load (no. of copies/ml)^c^	24,375 (509–925,557)	LDL (LDL-1170)	12,535 (381–945,989)	LDL (LDL-1044)
No (%) of children with the following WHO HIV staging				
1		22 (81)		22 (88)
2		1 (4)		
3	17 (63)	3 (11)	15 (60)	3 (12)
4	10 (37)	1 (4)	10 (40)	
Nasogastric tube no. (%)^d^	12 (44)	8 (30)	11 (44)	6 (24)
Formulation no. (%)				
*Suspension*	18 (67)	10 (37)	21 (84)	11 (44)
*Whole tablets*	7 (26)	14 (52)	4 (16)	11 (44)
*Crushed tablets*	2 (7)	3 (11)		3 (12)
Antiretroviral treatment no. (%)				
LPV/RTV-based regimen	16 (59)	16 (59)	15 (60)	14 (56)
EFV-based regimen	11 (41)	11 (41)	10 (40)	11 (44)
Antituberculosis treatment no. (%)				
Pyrazinamide	27 (100)		25 (100)	
Terizidone	26 (96)		24 (96)	
Ethambutol	25 (93)		23 (92)	
Ethionamide	25 (93)		23 (92)	
High-dose isoniazid	24 (89)		22 (88)	
Amikacin^e^	23 (85)		22 (88)	
Levofloxacin^f^	9 (33)		9 (36)	
Ofloxacin^f^	9 (33)		8 (32)	
Moxifloxacin^f^	7 (26)		6 (24)	
PAS	5 (19)		4 (16)	
Rifampicin^g^	4 (15)		4 (16)	
Linezolid	2 (7)		2 (8)	
Clofazimine	2 (7)		2 (8)	
Capreomycin^e^	1 (4)		1 (4)	

IQR, interquartile range; LDL, lower than detectable limit; LPV/RTV, lopinavir/ritonavir; EFV, efavirenz; PAS, *para*-aminosalisylic acid.

a A total of 54 HIV-infected children on lamivudine, of which 50 were also on abacavir were included in the study in two groups; 27 (25 on abacavir) HIV-infected children on MDR-TB treatment and 27 (25 on abacavir) HIV-infected non-TB controls.

b Weight- and height-for-age Z-scores of < -2 include underweight/stunted to severely underweight/stunted participants. 1990- British Z-scores were used.

c Most recent routine result within 6 months of the pharmacokinetic sampling day. Seven participants for the CD4^+^ T-cell counts and six participants for viral load have been excluded from the analysis.

d Some children (<2 years), that refused to swallow, were dosed using a nasogastric tube.

e Intramuscular injectable drugs.

f During 2012, levofloxacin and moxifloxacin replaced ofloxacin as fluoroquinolones of choice for MDR-TB treatment in South Africa.

g Of the participants receiving rifampicin, three where on an efavirenz-based regimen and one on a lopinavir/ritonavir-based regimen with super-boosted ritonavir. A possible effect of rifampicin in these patients were tested.

A total of 322 samples for lamivudine and 299 samples for abacavir were collected (2 lamivudine and one abacavir pharmacokinetic profile had only one pre-dose sample). Seven and 36 samples were below the LLOQ, for lamivudine and abacavir, respectively, and most of these (Zhang et al., 2012; World Health Organization, 2018) were the two predose samples. In 4 participants (3 of whom were out-patients) both drugs had LLOQ at all predose concentrations available (7 samples), while the pharmacokinetic profile observed after the supervised dose did not indicate low concentrations compatible with the undetectable trough concentration observed. For these patients, a missed evening dose was assumed. Another three samples were excluded due to values incompatible to the dose times of both drugs.

Antituberculosis drugs are listed in Table 1. Four children received rifampicin on the sampling day because of inconclusive or pending rifampicin susceptibility results; three were on an efavirenz- and one on a lopinavir/ritonavir-based regimen with super-boosted ritonavir. In addition, one participant, received rifampicin up to 3 days before the sampling day, and was on lopinavir/ritonavir-based regimen with super-boosted ritonavir. A possible delayed effect of rifampicin for this patient along with the other rifampicin patients were retained.

Creatinine levels were routinely obtained in 25/27 (93%) of the children in the MDR-TB group, and these all had values in the normal range. Creatinine levels for the HIV group were not done as it is not part of routine standard of care.

**FIGURE 1:**
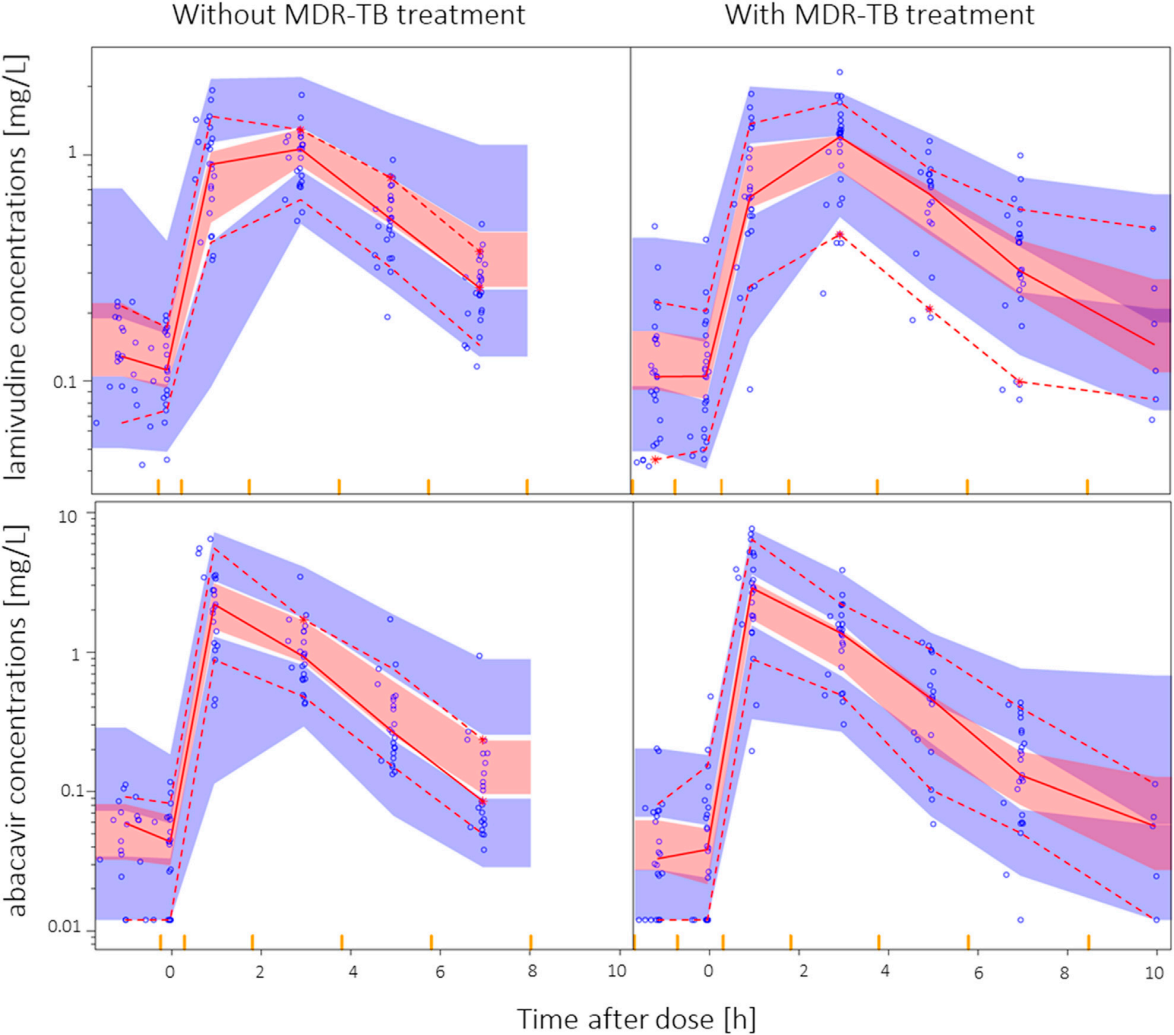
Visual predictive check for lamivudine and abacavir in HIV-positive children stratified by MDR-TB vs controls, using 1,000 simulations. The solid and dashed lines represent the fifth, 50th, and 95th percentiles of the observed data, while the shaded areas (pink and blue) are the model-predicted 95% confidence intervals for the same percentiles. Observed data are displayed as blue circles. Two children on once-daily dosing, sampled at later time points, were removed for the visual predictive check for display purposes.

A two-compartment model with first-order elimination and transit compartment absorption was found to suitably describe the pharmacokinetics of both lamivudine and abacavir. The final parameter estimates are presented in Table 2. A Visual Predictive Check plot stratified by MDR-TB treatment status is displayed in Figure 1. Goodness of fit plots for both models are presented in Supplementary Figure S1 (Jonsson and Karlsson, 1998).

**TABLE 2:** Parameter estimates of the final model for lamivudine and abacavir pharmacokinetics in HIV-positive children^a^.

Parameters	Lamivudine		Abacavir	
	Typical value (95% CI)	BSV^*^ or BOV** % (95% CI)	Typical value (95% CI)	BSV^*^ or BOV** % (95% CI)
CL (L/h)^b^	10.8 (9.89, 11.7)	14.7* (9.74, 20.5)	16.3 (14.5, 18.0)	17.7* (11.8, 22.7)
V_c_ (L)^b^	26.5 (24.0, 29.3)		21.7 (19.1, 24.1)	
k_a_ (1/h)	1.07 (0.878, 1.37)	66.0** (51.0, 83.0)	1.77 (1.49, 2.06)	74.9** (56.6, 93.9)
MTT–tablet (h)	0.643 (0.458, 0.852)	77.4** (54.5, 106)	0.458 (0.218, 0.772)	119** (89.6, 149)
Effect of suspension on MTT (%)	−46.3 (-67.3, -18.4)		−81.6 (−90.3, −63.1)	
NN ( )	6.38 (4.36, 9.01)		3.70 (2.70, 4.57)	
F ( )	1 (fixed)	29.4** (23.5, 36.3)	1 (fixed)	48.9** (40.2, 57.3)
Q (L/h)^b^	2.25 (1.83, 2.69)		1.42 (1.06, 1.75)	
V_p_ (L)^b^	69.8 (36.1, 106)		12.0 (9.09, 15.1)	
γ ( )^c^	3.32 (2.31, 4.68)		3.94 (2.72, 5.74)	
PMAGE_50_ (months from conception)^c^	10.6 (8.53, 12.6)		11.9 (9.54, 14.6)	
Additive error (mg/L)	0.0048 (fixed)^d^		0.0048 (fixed)^d^	
Proportional error (%)	10.8 (9.47, 12.6)		19.2 (16.8, 22.0)	

a Data are for 54 children on lamivudine, including 27 on treatment for Multidrug-resistant *tuberculosis* (MDR-TB) and 50 on abacavir, including 25 on MDR-TB treatment. 95% CI, 95% confidence interval as obtained by the sampling importance resampling (SIR) method; CL, apparent oral clearance; V_c_, apparent central volume of distribution; Q, apparent oral intercompartmental clearance; V_p_, apparent peripheral volume of distribution; k_a_, absorption rate constant; MTT, absorption mean transit time; NN, number of transit compartments; F, bioavailability; BSV, between-subject variability; BOV, between-occasion variability; PMAGE_**50**_ is the postmenstrual age at which 50% of the maturation is reached.

b All clearance and volume of distribution parameters are estimated by allometric scaling using body weight and the values reported here refer to a child with the median weight of 15 kg.

c Bayesian prior, with 20% uncertainty, from Bouazza et al. (Sy et al., 2015) for lamivudine and Rabie et al. (Garcia-Prats Donald et al., 2013) for abacavir were used for the estimation of maturation.

d The estimate of additive error for lamivudine and abacavir tended to 0, so it was fixed to 20% of the LLOQ for each drug.

The estimate of additive error for both lamivudine and abacavir was small and not robust and was therefore conservatively fixed to 20% of the LLOQ for each drug.

Allometric scaling was used to account for size differences and improved the model fit for both drugs (77 and 45 points decrease in −2LL for lamivudine and abacavir, respectively). Using fat-free mass instead of body weight did not provide any further benefit in terms of model fit. Maturation could be identified; clearance was predicted to reach half its mature value ∼ 2 months (lamivudine) and ∼ 3 months (abacavir) after birth, with both drugs being fully mature at ∼ 2 years of age. Since the maturation parameters could not be identified precisely, Bayesian priors (Gisleskog et al., 2002) based on reports from larger comparable populations (Bouazza et al., 2011; Rabie et al., 2020) with 20% uncertainty was used to stabilize the models. The typical clearance in a 15 kg child was estimated at 10.8 L/h for lamivudine and 16.3 L/h for abacavir.

The mean absorption transit time for the suspension formulation of both drugs were significantly faster than tablets (21 vs 39 min for lamivudine and 5 vs 27 min for abacavir), but no difference was found for bioavailability.

No significant differences were detected due to lopinavir/ritonavir-, ritonavir superboosted-vs efavirenz-containing ART, sex, or the use of a nasogastric tube. Exclusion of the participants on rifampicin also did not affect our findings.

MDR-TB treatment was not found to significantly affect the pharmacokinetics of lamivudine or abacavir. Terizidone, ethambutol, ethionamide, high-dose isoniazid, pyrazinamide and amikacin were tested individually with similar results. None of the models including effects on clearance, bioavailability, or absorption rate constant achieved a significant improvement in −2LL. The visual predictive checks stratified by MDR-TB group vs controls are shown in Figure 1, showing that a model assuming no effect of MDR-TB treatment was suitable for both datasets.

Two participants in the control group remained on once-daily lamivudine and abacavir dosing. The pre-sampling dose was therefore not observed. As precaution, we excluded these participants in the model development process and when evaluating TB treatment as covariate, but this made no difference, so the patients were retained in the final model.

The *a posteriori* power calculations predicted that, at a significance level of *p* < 0.01, our study design was expected to be able to detect with 80% power a 20% decrease in lamivudine exposure (i.e. 20% increase in clearance) in the MDR-TB group. For abacavir, at the same power and significance level, a 25% decrease in exposure (i.e. 25% increase in clearance) would be detected. Therefore, if a difference greater than these effects were present in the data, our model would have an 80% chance of detecting it at *p* < 0.01.

## Discussion

We describe the pharmacokinetics of lamivudine and abacavir in HIV-positive children with and without MDR-TB treatment and could detect no significant DDIs of MDR-TB treatment on lamivudine and abacavir. The newer antituberculosis drugs, such as bedaquiline and delamanid were not yet available for the treatment of MDR-TB in children during the time of the study, and were therefore not included. Despite modest numbers, our results are reassuring for the antituberculosis drugs used commonly in children including terizidone, ethambutol, ethionamide, high-dose isoniazid, pyrazinamide, amikacin and fluoroquinolones as a group. For other single drugs, such as moxifloxacin, levofloxacin, linezolid and PAS, definitive conclusions were not possible given our small numbers.

The estimated value for lamivudine oral clearance of 10.8 L/h was similar to previously published reports in HIV-positive children (infants to 18 years) (Tremoulet et al., 2007; Bouazza et al., 2011; Zhang et al., 2012; Piana et al., 2013). The median allometrically scaled lamivudine clearance ranged from 7.2–16.5 L/h in four studies (*n* = 752) with similar populations. For abacavir, the estimated value for oral clearance of 16.3 L/h was similar to previously published reports in HIV-positive children (infants to 16 years) (Jullien et al., 2006; Zhao et al., 2013; Rabie et al., 2020). The median allometrically scaled abacavir clearance ranged from 14.7–17.8 L/h in three studies (*n* = 261) with similar populations. Overall, findings from these studies on both drugs were compatible with our data.

Abacavir’s bioavailability has been shown to decrease during co-treatment with rifampicin and lopinavir/ritonavir (Rabie et al., 2020). Our study did not find this likely due to the small number of children receiving rifampicin, and due to the rest of the children being on an efavirenz-containing regimen.

Our study has several limitations, the main one being the observational nature, where children were on individualized MDR-TB treatment regimens with varying combinations of antituberculosis drugs, which increased the possibility of unknown confounders.

Despite our modest sample size, we believe our findings to remain valuable, since large clinically relevant effects would have been detected. A cross-over design may have increased the power of the analysis, but this was not feasible in our study.

Another limitation is that actual routine creatinine clearance samples were not available for all participants, and therefore we were unable to test renal function as covariate. This would have been an important covariate in the model for lamivudine since it is primarily renally eliminated. However, all children in the MDR-TB group, who are generally sicker than the controls, had normal renal function.

In conclusion, we found no significant effect on key pharmacokinetic parameters (clearance, bioavailability and absorption) of lamivudine and abacavir when co-administered with antituberculosis drugs commonly used for MDR-TB treatment in HIV-positive children. While a modest-sized study, our findings are reassuring. Optimal and safe dosing of both ARVs and MDR-TB treatment in HIV-co-infected children is essential. Additional research is needed to evaluate DDIs between ARVs and increasingly used TB drugs including moxifloxacin, levofloxacin, clofazimine, linezolid, bedaquiline and delamanid.

## Data Availability

The data that support the findings of this study are available from the corresponding author upon reasonable request.
