# Supplementary figures and images for: Pharmacokinetics and Drug-Drug Interactions of Abacavir and Lamuvudine Co-administered With Antituberculosis Drugs in HIV-Positive Children Treated for Multidrug-Resistant Tuberculosis
Source: Front Pharmacol. 2021 Oct 8;12:722204. doi: 10.3389/fphar.2021.722204 (PMC8531271; doi:10.3389/fphar.2021.722204)

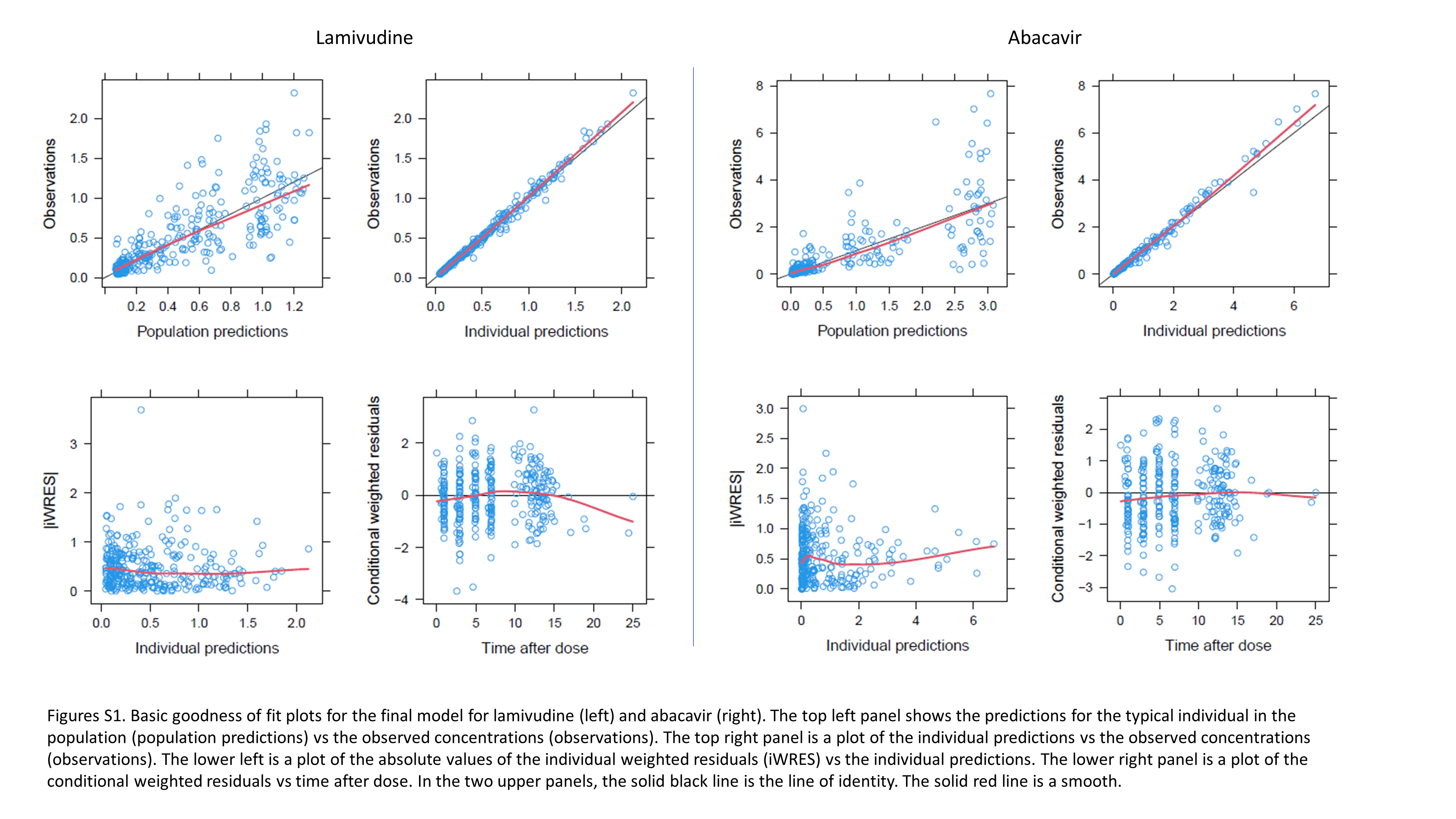

Supplement: Supplementary file 1 [file Image1.tif]
